# Pre-Absorbed Immunoproteomics: A Novel Method for the Detection of *Streptococcus suis* Surface Proteins

**DOI:** 10.1371/journal.pone.0021234

**Published:** 2011-06-21

**Authors:** Wei Zhang, Guangjin Liu, Fang Tang, Jing Shao, Yan Lu, Yinli Bao, Huochun Yao, Chengping Lu

**Affiliations:** Key Laboratory of Animal Disease Diagnostic & Immunology, Ministry of Agriculture, Nanjing Agricultural University, Nanjing, China; Wageningen University and Research Centre, The Netherlands

## Abstract

*Streptococcus suis* serotype 2 (SS2) is a zoonotic pathogen that can cause infections in pigs and humans. Bacterial surface proteins are often investigated as potential vaccine candidates and biomarkers of virulence. In this study, a novel method for identifying bacterial surface proteins is presented, which combines immunoproteomic and immunoserologic techniques. Critical to the success of this new method is an improved procedure for generating two-dimensional electrophoresis gel profiles of *S. suis* proteins. The *S. suis* surface proteins identified in this study include muramidase-released protein precursor (MRP) and an ABC transporter protein, while MRP is thought to be one of the main virulence factors in SS2 located on the bacterial surface. Herein, we demonstrate that the ABC transporter protein can bind to HEp-2 cells, which strongly suggests that this protein is located on the bacterial cell surface and may be involved in pathogenesis. An immunofluorescence assay confirmed that the ABC transporter is localized to the bacterial outer surface. This new method may prove to be a useful tool for identifying surface proteins, and aid in the development of new vaccine subunits and disease diagnostics.

## Introduction

*Streptococcus suis* is a swine pathogen that can cause meningitis, pneumonia, septicemia, and arthritis in animals [1]. As a zoonotic agent, *S. suis* can also be transmitted to humans that come into contact with infected pigs or pork-derived products, and infection can lead to fever, nausea and vomiting [2], meningitis, endocarditis, and septic shock [3], [4]. Among the 33 serotypes of *S. suis*, serotype 2 (SS2) is the most virulent and prevalent serotype found in diseased pigs. The mechanisms of *S. suis* pathogenesis are still not well understood [1], and this hampers efforts to develop effective vaccines and treatments.

Surface proteins of pathogenic bacteria can serve as protective antigens and virulence markers, though they can be technically challenging to identify. Several biochemical and microbiological techniques have been employed to characterize bacterial surface proteins, including multidimensional protein identification [5], stable isotope labeling [6], biotinylation approaches [7], surface shaving approaches [8], genome analyses, and protein and antibody arrays [9]. During the last decade, immunoproteomics has become an increasingly popular method used for identifying immunoreactive proteins. This technique involves the separation of proteins by two-dimensional electrophoresis (2-DE) and Western blotting. Though host antibodies primarily recognize proteins on the surface of a bacterium, non-surface proteins can also become immunogenic after proteolytic digestion in host antigen presenting cells (APCs). Thus, distinguishing between antibodies that recognize surface and non-surface proteins is an important consideration when designing immunoproteomics experiments to identify potential vaccine candidates.

**Figure 1:**
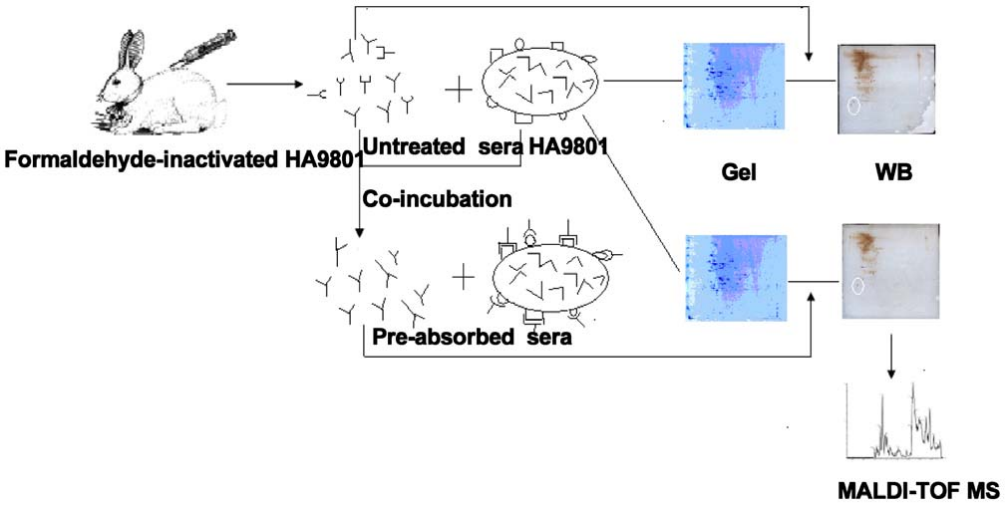
Schematic diagram of the surface protein detection assay. One sample of the *S. suis*-derived antiserum was pre-absorbed with whole-cell *S. suis* to remove the antibodies that recognize outer surface proteins (“pre-absorbed”). Then, untreated and “pre-absorbed” antisera were used to probe 2-DE gels of *S. suis* proteins. Spots that appear in the blot probed with untreated antiserum, but that were absent from the blot probed with “pre-absorbed” antiserum, were evaluated to identify the surface proteins.

Cross-absorption is a powerful method used in conventional serological techniques to minimize cross reaction during agglutination [10]. The serum cross-absorption process was modified to remove antibodies that recognized bacterial surface antigens and this produced novel “pre-absorbed” antiserum. We used untreated and “pre-absorbed” antisera to probe 2-DE blots of *S. suis* cell lysates. Protein spots that appeared in the blot probed with untreated serum, but that were absent in the blot treated with pre-absorbed serum, were assumed to be surface proteins. These proteins were identified using matrix-assisted laser desorption ionization–time of flight mass spectrometry (MALDI-TOF MS). We used bioinformatics predictions and immunofluorescence to verify that the proteins identified were located on the bacterial cell surface. A schematic diagram of the surface protein detection method is shown in Figure 1.

In this present study, we demonstrate that our immunoproteomic-based approach can detect bacterial surface proteins. Indeed, we identified two SS2 surface proteins from *S. suis*, including muramidase-released protein precursor (MRP), which is known to be a strong SS2 virulence factor [11], [12], and an ABC transporter protein that we show is located on the bacterial cell surface. Our method may prove useful for the development of new vaccine subunits and disease diagnostics.

## Results and Discussion

A novel method that combined immunoproteomic and immunoserologic approaches was established for identifying bacterial cell surface proteins. Using this method, two surface proteins of *S. suis* were identified, demonstrating the utility of this approach for studying bacterial outer surface proteins.

**Figure 2:**
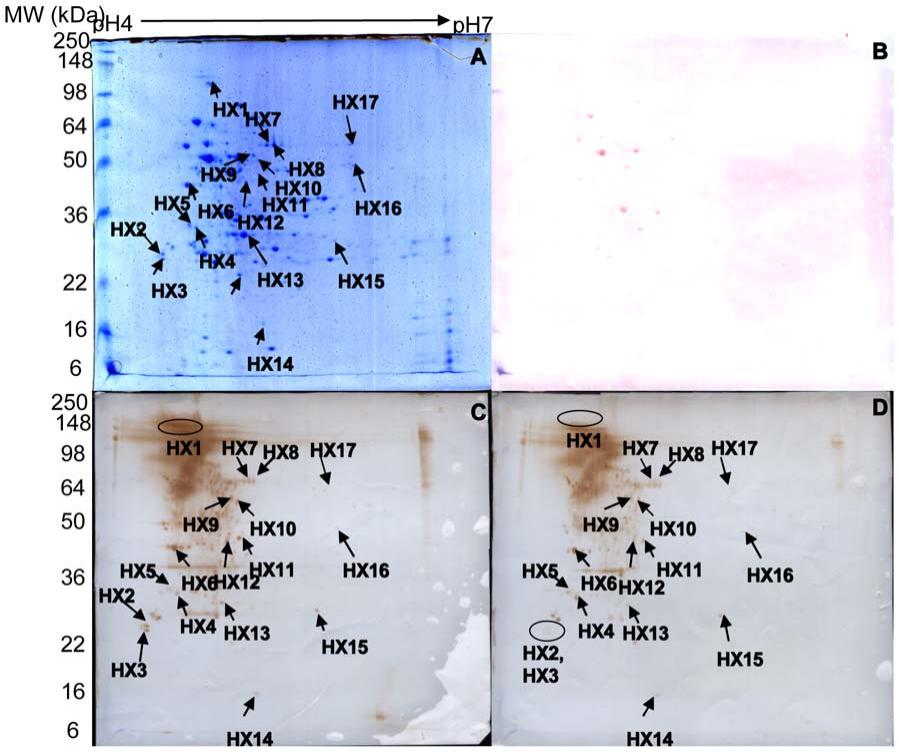
2-DE gel and Western blot analyses of HA9801. (A) HA9801 total cell proteins (pH 4–7, 13 cm), stained with colloidal Coomassie brilliant blue G-250. (B) 2-DE blot of *S. suis* stained with Ponceau S. (C) 2-DE blot of *S. suis* proteins probed with untreated antiserum. (D) 2-DE blot of *S. suis* proteins probed with “pre-absorbed” antiserum.

The sample preparation of proteins for 2-DE analyses was improved, and better 2-DE profiles were obtained than previously [13], [14], [15]. Proteomic-based approaches for investigating *S. suis* have been hampered by the preparation of protein samples. Mutanolysin, which is purified from the culture supernatant of *Streptomyces globisporus*, is an efficient reagent that can be used to obtain protoplasts of *Streptococcus mutans* [16], [17], as it is highly effective for inducing the lysis of bacterial cells without any associated proteolytic activity [18]. The small quantity of mutanolysin used in these experiments was not visible on the 2-DE gels and did not influence the protein profiles [19], [20]. Based on previous results from our lab and other workers [13], [21], mutanolysin was used to generate *S. suis* protoplasts, which subsequently were disrupted by sonication. This yielded bacterial proteins for 2-DE separation analyses by isoelectric point (pI) and pH (range 4–7). The 2-DE separation profiles were stained with colloidal Coomassie brilliant blue G-250 reagent and these are shown in Figure 2A. Ponceau S-stained membranes revealed that only very few proteins were found in high abundance (Figure 2B).

### Western blots of the 2-DE

An initial cross-absorption step to produce “pre-absorbed” serum eliminates cross antigens and ensures the specificity of slide agglutination and ring precipitation [10]. Gu *et al.* [22] cross-adsorbed whole bacterial cells and bacterial cell lysates with convalescent serum to eliminate antibodies reactive with in vitro-expressed antigens. This approach allows for the identification of in vivo-expressed protein antigens that are upregulated during *S. suis* infection. Thus, the removal of antibodies that recognize *S. suis* outer surface proteins from antiserum using intact bacteria prior to blotting could provide a means of differentiating outer and cytoplasmic bacterial proteins in an immunoproteomic analysis. So in this present study, Western blots of the 2-DE protein gels were probed with either untreated or the “pre-absorbed” sera. Probing with the untreated serum identified many immune-reactive protein spots (Figure 2C). However, when an identical blot was probed using the “pre-absorbed” serum, some of the spots, such as HX1, HX2 and HX3, were less distinct or had disappeared (Figure 2D). This experiment was repeated in triplicate, and the separation profiles were consistent and highly reproducible.

### Identification of immunoreactive proteins by bioinformatic analyses

In total, 17 immune-reactive protein spots were excised from preparative 2-DE gels, subjected to tryptic digestion, and then analyzed by MALDI-TOF MS. The top ranking protein identified in each spot and their predicted subcellular locations are listed in Table S1.

Spot HX1 contained MRP (gi|253753453), which is an important protective antigen in SS2. The HX1 spot in the 2-DE blot probed with untreated serum was much darker than the HX1 spot in the blot probed with “pre-absorbed” serum. This indicates that the amount of antibody against MRP in “pre-absorbed” serum was decreased by the absorption process. Spots HX2 and HX3 contained the same protein, specifically a periplasmic ABC transporter protein (gi|146319723), which is a new immunoreactive protein from *S. suis* identified for the first time. The small differences in pI and molecular weight values of the two spots on the 2-DE gel are likely to be due to slight protein modifications.

Table 1 shows the predicted transmembrane regions and subcellular localizations of the different proteins according to three software programs, namely PSORTb v.3.0.0, LocateP and Gpos-mPLoc. The location of the ABC transporter was unknown according to the PSORT server. However, a recent study showed that PSORT does not detect lipoprotein motifs and assigns low probability scores to the topological organization of proteins with transmembrane regions, especially in Gram-positive bacteria [23]. Moreover, PSORT results are based on the annotation of the data in the Swiss-Prot database [24], and consequently inconsistencies are inherited from those designations. The LocateP program [25] predicts the localization of bacterial proteins to one of seven compartments within Gram-positive bacteria: intracellular, multi-transmembrane, N-terminally membrane anchored, C-terminally membrane anchored, lipid-anchored, LPXTG-type cell-wall anchored, and secreted/released proteins. Furthermore, the Gpos-mPLoc software [26], originally developed for identifying the subcellular localization of Gram-positive bacterial proteins by fusing information on gene ontology, can be used to predict the subcellular location of a query protein to one of four sites: cell membrane, cell wall, cytoplasm, and extracellular. Therefore, LocateP and Gpos-mPLoc were the preferred primary references used for subcellular protein location predictions. LocateP predicted that HX2 is a lipid-anchored protein located on the outer membrane, while Gpos-mPLoc predicted that HX2 is located on the cell wall.

**Table 1:** The predicated subcellular location of differential proteins.

Spot no.	Protein Identified	The score of PSORTb version 3.0.0 programs					LocateP	Gpos-mPLoc
		Cytoplasmic	Cytoplasmic Membrane	Cellwall	Extracellular	Final Prediction		
HX1	muramidase-released protein precursor	0.00	0.00	10.00	0.00	Cellwall	Cellwall	Extracell
HX2	amino acid ABC transporter periplasmic protein	0.00	3.33	3.33	3.33	Unknown	Extracellular	Cellwall
HX3	amino acid ABC transporter periplasmic protein	0.00	3.33	3.33	3.33	Unknown	Extracellular	Cellwall

Many other significant proteins were identified that localized in the cytosol. For instance, we identified that pyruvate kinase (HX7, HX8), a key cytosolic regulatory enzyme in the glycolytic pathway, was also found in the immunoproteomic study of *Streptococcus suis* serotype 9 [27]. Eight new *S. suis* immunoreactive proteins (spots HX5, HX9–13, HX16) are reported here for the first time. The protein in spot HX5 was identified to be the molecular chaperone DnaK, which promotes protein folding, interaction and translocation in response to stress, by binding to unfolded polypeptide segments. DnaK is a major antigen in *Streptococcus pneumoniae* [28], and is associated with the pathogenesis of this bacterium in pneumonia and meningitis [29]. The protein in spot HX11 shares homology with an ADP glucose pyrophosphorylase, an enzyme that is required for the biosynthesis of bacterial intracellular polysaccharide. This protein is associated with cavity formation by *S. mutans* [30], and biofilm formation and virulence in *Salmonella enteritidis* [31].

### Western blotting of recombinant proteins

**Figure 3:**
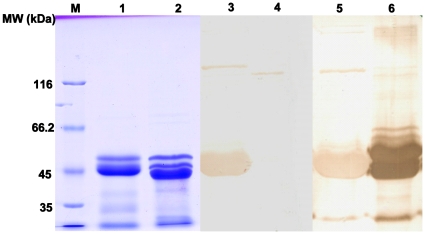
SDS-PAGE and Western Blot analysis of recombinant HX2 and HX1. SDS-PAGE analysis of purified rHX2 (lane 1) and rHX1 (lane 2) from the induced producer *E. coli* strains; Western blot analysis of rHX2 (lane 3) and rHX1 (lane 4) with hyperimmune sera against T15; Western blot analysis of rHX2 (lane 5) and rHX1 (Lane 6) with hyperimmune serum against HA9810; molecular size markers in kDa (Lane M).

To verify the subcellular location of HX2, HX1 and HX2 were expressed recombinantly. Sodium dodecyl sulfate polyacrylamide gel electrophoresis (SDS-PAGE) revealed that recombinant HX1 (rHX1) and HX2 (rHX2) were approximately 45 kDa (Figure 3, lanes 1 and 2). Western blot analysis revealed that both recombinant proteins were immunoreactive, but rHX2 reacted more weakly than rHX1 with the antiserum derived from *S. suis* HA9801 (Figure 3, lanes 5 and 6). This was consistent with the earlier immunoproteomics data. In addition, the serum against an avirulent *S. suis* strain (T15) did not react with rHX1 (MRP), but it did react with rHX2 (Figure 3, lanes 3 and 4), thus proving that T15 was not expressing the MRP protein [11]. Interestingly, the genome of T15 contains a similar MRP gene (unpublished observation), but the lack of expression is probably due to gene insertions or deletions in the operon.

### Subcellular location of HX2 on HA9801 and rHX2 binding of HEp-2 cells

**Figure 4:**
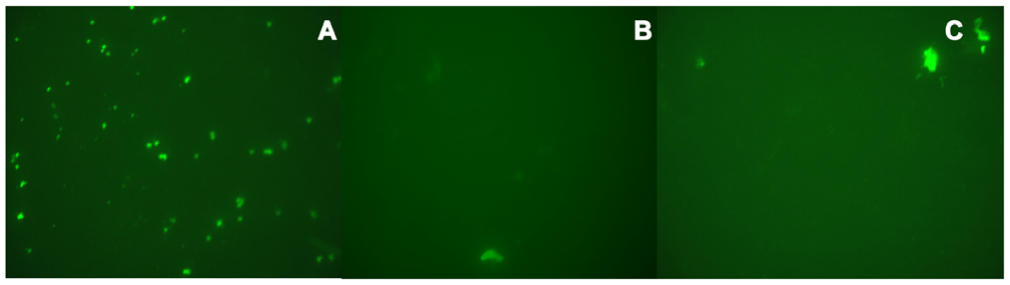
Analysis of HX2 outer surface localization on *S. suis* HA9801. Oil immersion fluorescence microscopy (×1,000) was conducted on *S. suis* HA9801 bacteria labeled with rHX2 antiserum (panel A), control rabbit serum (panel B), and PBS negative control (panel C); IgG-FITC was the secondary antibody.

**Figure 5:**
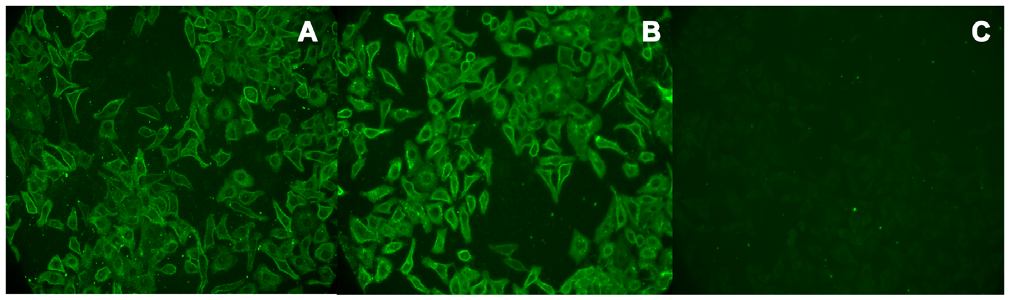
Adherence of rHX1 and rHX2 to HEp-2 cells. HEp-2 cells were incubated with (A) rHX2, (B) rHX1, and (C) PBS-BSA (negative control), and after washing, cells were incubated with rabbit antiserum to rHX2, antiserum to rHX1 and PBS-BSA, respectively. Then, HEp-2 cells were labeled with anti-rabbit IgG-FITC and examined by fluorescence microscopy.

ABC transporters are associated with virulence in bacteria. They import various nutrients required for survival in different niches and export substances toxic to the cell [32]. Lipid-anchored proteins are involved in a large variety of functions, and these include adhesins, transporters, receptors, enzymes, and virulence factors [33]. In order to identify whether HX2 from our immunoproteomic analysis resides on the outer surface of *S. suis*, whole-cell HA9801 bacteria were labeled with antiserum to rHX2, and HX2 was indirectly detected using fluorescein-conjugated goat anti-rabbit IgG (IgG-FITC). A strong fluorescent signal was detected on the outer surface of the HA9801 cells (Figure 4), confirming that HX2 is located on the outer surface and is in support of the bioinformatic prediction. Then, an indirect immunofluorescence assay was used to determine whether rHX2 adhered to HEp-2 cells. Green fluorescence was detected on HEp-2 cells pre-incubated with rHX2, whereas greater fluorescence intensity was seen on cells pre-incubated in rHX1, while no fluorescence was observed using bovine serum albumin in phosphate-buffered saline (PBS-BSA) (Figure 5). Thus, this transporter may be involved in the pathogenesis of *S. suis* by facilitating bacterial attachment to host cells.

### Distribution of HX2 gene among *Streptococcus* strains

Most *S. suis* strains examined gave the expected fragment size of approximately 750 bp of DNA per reaction mixture confirming the presence of the HX2 gene, while no amplification product was observed in another *Streptococcus* sp. (Table S2). Further, the gene encoding HX2 is present in diverse *S. suis* strains, and its distribution is unrelated to any particular serotype.

In conclusion, a novel immunoproteomics-based method was developed for detecting bacterial surface proteins. This method was used to detect surface proteins of *S. suis* with the goal of identifying proteins that could serve as potential vaccine candidates. Many experimental conditions were tested in order to separate proteins by 2-DE in attempts to identify greater numbers of outer surface antigens, including a pH range of 3–10 and Silver Staining; however, the initial results were unsatisfactory. Thus, the method presented herein will continue to be modified and improved to expand the range of surface proteins that can be detected.

## Materials and Methods

### Bacterial strains and culture conditions

The SS2 strain (*S. suis* HA9801) was isolated in 1998 [34]. *S. suis* T15, the avirulent SS2 strain, was kindly donated by Dr H.E. Smith (DLO-Institute for Animal Science and Health, The Netherlands) [35]. All the strains were grown in Todd Hewitt broth (THB; pH 7.8; BD Inc.) at 37°C.

### Protein sample extraction

Protein precipitations were performed according to Winterhoff [13], but with some modifications. Briefly, exponential-phase bacterial cultures were centrifuged at 10,000×g for 15 min at 4°C, and washed twice in PBS. Pellets were resuspended in buffer (Tris-HCl, MgCl_2_, 50% sucrose) that contained 1,000 U/mL mutanolysin (Sigma), and incubated for 90 min at 37°C. Then the spheroplasts were collected, resuspended in solution B (7 M urea, 2 M thiourea, 4% 3-[(3-cholamidopropyl)dimethylammonio]-1-propanesulfonate [CHAPS], and 65 mM dichlorodiphenyltrichloroethane [DTT] ; GE Healthcare) and sonicated in an ice bath for 50 cycles (5 s on at 100 W, followed by 10 s off). After 30 min incubation at 25°C, unbroken cells were removed by centrifugation at 10,000×g for 15 min at 4°C. Proteins in the supernatant were precipitated in 10% pre-chilled trichloroacetic acid (TCA) and incubated in ice-water for 30 min. After centrifugation at 10,000×g for 10 min at 4°C, the pellet was resuspended in 10 mL of pre-chilled acetone and washed twice. The final pellet was dried in air.

### Isoelectric focusing

Isoelectric focusing (IEF) was performed using an Ettan IPGphor-3 IEF system (GE Healthcare) and 13 cm gel strips (Immobiline DryStripk, pH 4–7; GE Healthcare). Prior to rehydration, the precipitated proteins were treated using a 2-DE Clean-up kit (GE Healthcare) to remove contaminants that can interfere with electrophoresis. Immobilized pH gradient (IPG) strips were rehydrated overnight at room temperature (RT) using rehydration solution (7 M urea, 2 M thiourea, 2% CHAPS, 0.2% DTT, 0.5% IPG buffer [pH 4–7], and 0.002% bromophenol blue). Each strip was loaded with 200 µg of protein, and IEF was carried out at 20°C for 12 h (maximum voltage of 8,000 V and maximum current of 50 µA per IPG strip; total 28,000 Vh).

### 2D SDS-PAGE

Prior to 2D SDS-PAGE, each IPG strip was washed in equilibration buffer 1 (375 mM Tris-HCl [pH 8.8], 6 M urea, 2% SDS, 2% DTT) for 15 min, followed by equilibration buffer 2 (375 mM Tris-HCl [pH 8.8], 6 M urea, 2% SDS, 2.5% iodoracetamide) for 15 min. Each IPG strip plus an SDS-PAGE molecular weight standard (Invitrogen) was loaded onto a homogeneous 12% polyacrylamide gel and sealed with 1% agarose. Electrophoresis was performed at 15°C using an initial voltage of 110 V for 30 min, followed by 220 V until the tracking dye had reached the bottom of the gel. All gels were stained with colloidal Coomassie brilliant blue G-250 according to the manufacturer's instructions (GE Healthcare). Each 2D IEF/SDS-PAGE experiment was repeated three times.

### Preparation of hyperimmune sera

Rabbits were first determined to be negative for *S. suis* antibodies using an indirect enzyme-linked immunosorbent assay (ELISA) developed in-house. Subsequently, these rabbits were immunized with formaldehyde-inactivated *S. suis* HA9801 and T15 bacteria, using Montanide ISA 206 VG (SEPPIC Co. Ltd) as the adjuvant. Two doses of 1.0×10^9^ cells/rabbit were administered by intramuscular injections at 3-week intervals. Sera from immunized and control rabbits were collected before the first and after the second immunizations. The titers of the sera were evaluated using indirect ELISA.

### Preparation of “pre-absorbed” sera

The absorption protocol used was as described by Mittal et al. [10], [22]. Briefly, exponential cultures of *S. suis* HA9801 were centrifuged at 3,000×g for 15 min at 4°C, and then washed twice in PBS. A total of 1.0×10^8^ bacteria were suspended in 100 µl of HA9801 hyperimmune serum, incubated for 2 h at 37°C, and then overnight at 4°C. Bacteria were pelleted by centrifugation at 10,000×g for 30 min. The supernatant was collected and used for Western blotting as the “pre-absorbed” serum.

### Western blotting

Protein samples from each SDS-PAGE gel were transferred onto polyvinylidene fluoride (PVDF) membranes (GE Healthcare) for 2 h at 0.65 mA/cm^2^ using a semi-dry blotting apparatus (TE77, GE Healthcare). Membrane-bound proteins were detected by staining with Ponceau S [36]. For this, the PVDF membrane was submerged in the stain solution, containing 0.1% Ponceau S (Solarbio) (w/v) and 5% acetic acid (v/v), with gentle agitation for 5 min. The membrane was washed several times with dH_2_O until the protein bands were visible, and then digitally scanned using a Umax scanner (GE Healthcare). Subsequently, the Ponceau S stain was removed from the membranes by rinsing gently in dH_2_O. After removing Ponceau S, the membrane was blocked with 5% (w/v) skim milk in 50 mM Tris-HCl buffer (pH 7.4) containing 0.05% Tween 20 (TBST) for 2 h at RT. Then, the blocked membrane was incubated with HA9801 hyperimmune serum or HA9801 “pre-absorbed” serum (1∶5,000 dilution) for 2 h at RT, followed by three washes with TBST for 10 min each wash. The membrane was incubated with horseradish peroxidase-goat anti-rabbit serum (Boster; 1∶10,000 dilution) for 1 h at RT, washed three times with TBST, and then developed by adding 3,3′-diaminobezidine (Tiangen Co. Ltd) until optimal color was obtained. Western blotting was performed in triplicate.

### MALDI-TOF MS and database searching

Proteins identified from the 2-DE blots as potential surface proteins were excised from duplicate SDS-PAGE gels and used for in-gel trypsin digestion and MALDI-TOF MS (TOF Ultraflex II mass spectrometer, Bruker Daltonics). Peptide mass fingerprinting (PMF) data were analyzed using the MASCOT server (http://www.matrixscience.com). MASCOT searches were used to determine which peptides were to be considered significant and used for the combined peptide scores. The extent of sequence coverage, number of matched peptides, and the score probability obtained from the PMF data were all used to validate protein identifications. Low-scoring proteins were either verified manually or rejected.

### Bioinformatics analysis

Sequences of the identified proteins were searched using the BLASTX server (http://www.ncbi.nlm.nih.gov/BLASTX/) to find homologous sequences. The PSORT server (http://www.psort.org/), LocateP (http://www.cmbi.ru.nl/locatep-db) and Gpos-mPLoc (http://www.csbio.sjtu.edu.cn/bioinf/Gpos-multi/) programs were used to predict subcellular localizations for the proteins.

### Expression of recombinant proteins

Genes encoding proteins identified as potential surface proteins were amplified by PCR from genomic DNA of *S. suis* HA9801 using the primers listed in Table 2. PCR products were cloned into the pET-28a or pET-32a expression vectors, and the resulting plasmids were used to transform *Escherichia coli* BL-21. Transformed cells were grown at 37°C until the OD_600_ reached 0.3, at which point protein expression was induced by adding 1 mM isopropyl-β-D-thiogalactopyranoside for 4–5 h at 37°C. Cells were harvested by centrifugation at 10,000×g for 10 min at 4°C. Protein purification was performed using Ni-Trap™ columns (GE Healthcare). Purified recombinant protein was analyzed by SDS-PAGE stained with Coomassie brilliant blue R-250, or transferred to PVDF membranes and probed using the HA9801 or T15 hyperimmune rabbit sera.

**Table 2:** Primer sequences used for cloning immunoreactive genes candidates.

Spot no.	Primer sequence used for clone	Length of PCR products	expression vector
HX1	Forward: 5-CGAGAATTCTGCTGAAAATACGAGTGC	970 bp	pET-30a
	Reverse: 5-TATCTCGAGCTATGCCACATAATCATACCC		
HX2	Forward: 5-TGAGAATTCGCCTGCAATTCATCTGCA	750 bp	pET-28a
	Reverse: 5-CCGCTCGAGTTACTTAGCTTTTGATACG		

### Antisera to recombination proteins

Purified recombinant proteins were used to immunize rabbits and Montanide ISA 206 VG (SEPPIC Co., Ltd) at 0.5 mg/mL was used as adjuvant. The proteins were administered by intramuscular injections three weeks apart. The titers of sera were evaluated using indirect ELISA.

### Subcellular location of HX2 in HA9801 cells

*S. suis* HA9801 was grown overnight in THB at 37°C, then centrifuged at 10,000×g for 5 min at 4°C, and washed twice in PBS. Bacteria were diluted in PBS to 5×10^7^ cell/mL. To the wells of an immunofluorescence slide (Cel-line) were added 10 µL of the bacterial suspensions, while PBS was used as the negative control. Slides were dried in air, fixed for 10 min in cold acetone and stored at −70°C until required. Antisera to purified recombinant protein or control rabbit antisera were added to each well (1∶200 dilution) and incubated at 37°C for 2 h. The slide was washed three times with PBS, and then goat anti-rabbit IgG-FITC (1∶200 dilution; Boster) was added to each well. Wells were incubated for 1 h at 37°C, and then the wash procedure was repeated [37]. Fluorescence was detected microscopically (ZEISS, Germany).

### Cell culture methods

HEp-2 cells (ATCC CCL 23) were maintained in modified Eagle's medium supplemented with 10% fetal bovine serum (GIBCO). Cells were incubated at 37°C in a humidified 5% CO_2_ incubator. For all experimental assays, 24-well tissue culture trays (TPP) were seeded with 5.0×10^4^ HEp-2 cells/mL. Prior to the assays, semi-confluent monolayers were washed using PBS, dried in air and fixed in 4% paraformaldehyde for 1 h at RT. To each well was added 30 mM aminoacetic acid for 5 min, followed by 0.1% Triton X-100 (Sigma) for a further 5 min. After three washes with PBS, the wells were blocked with 3% PBS-BSA (GIBCO) overnight at 4°C, and then washed prior to use.

### Indirect immunofluorescence assay on HEp-2 cells

Purified recombinant proteins, rHX1 and rHX2, were diluted in PBS-BSA to 10 ng/mL and added to the HEp-2 monolayers in 24-well cell culture trays, and then incubated at 37°C for 2 h. PBS-BSA was used as control. Wells were washed three times with PBS. Antisera to each recombinant protein (1∶200 dilution in PBS-BSA) were added to the corresponding wells and incubated at 37°C for 2 h [38]. Then, wells were incubated for 1 h at 37°C with anti-rabbit IgG-FITC (1∶200 dilution in PBS-BSA; Santa). After a final wash, wells were observed using the fluorescence microscope.

## Supporting Information

Table S1 **Summary of analysis performed on protein spots identified by MALDI-TOF MS.**
(DOC)

Table S2 **Distributions of the** ***HX2*** **gene in** ***Streptococcus sp.*** **Strains.** ^a^The serotypes of SS strains were confirmed by the agglutination test. N, nontypeable.
(DOC)
