# Supplementary material for: Pre-Absorbed Immunoproteomics: A Novel Method for the Detection of Streptococcus suis Surface Proteins
Source: PLoS One. 2011 Jun 21;6(6):e21234. doi: 10.1371/journal.pone.0021234 (PMC3119691; doi:10.1371/journal.pone.0021234)
Supplement: Table S1 — Summary of analysis performed on protein spots identified by MALDI-TOF MS. (DOC) [file pone.0021234.s001.doc]

**Supporting Information**

**Table S1. Summary of analysis performed on protein spots identified by MALDI-TOF MS**

| **Spot no.** | **Gi Number** | **Protein Identified** | **Theoretical MW/pI** | **Experimental**  **MW/pI** | **Protein subcellular location prediction** | | |
| --- | --- | --- | --- | --- | --- | --- | --- |
|  |  |  |  |  | **PSORTb 3.0.0** | **LocateP** | **Gpos-mPLoc** |
| HX1 | [gi|253753453](http://www.cmbi.ru.nl/locatep-db/cgi-bin/locatepdb.py?organism=Streptococcus_suis_P1_7&gi=GI:253753453) | muramidase-released protein precursor (136 kDa surface protein, *streptococcus suis* P1/7) | 135713/4.87 | 138 000/4.78 | Cellwall | Cellwall | Extracell |
| HX2 | [gi|146319723](http://www.matrixscience.com/cgi/protein_view.pl?file=../data/20090925/FtirIxsaT.dat&hit=1) | amino acid ABC transporter periplasmic protein[*Streptococcus suis* 05ZYH33] | 28609/4.72 | 28000/4.47 | Unknown | Extracellular | Cellwall |
| HX3 | [gi|146319723](http://www.matrixscience.com/cgi/protein_view.pl?file=../data/20090925/FtirIxsaT.dat&hit=1) | amino acid ABC transporter periplasmic protein[*Streptococcus suis* 05ZYH33] | 28609/4.72 | 26000/4.47 | Unknown | Extracellular | Cellwall |
| HX4 | gi|11612452 | elongation factor Tu [*Streptococcus suis*] | 29603/4.54 | 33000/4.75 | Extracellular | Cytoplasmic | Cytoplasm |
| HX5 | gi|146317956 | molecular chaperone DnaK [*Streptococcus suis* 05ZYH33] | 64787/4.62 | 34000/4.7 | Unknown | Cytoplasmic | Cytoplasm |
| HX6 | gi|146319787 | sugar ABC transporter substrate-binding protein [*Streptococcus suis* 05ZYH33] | 44245/4.63 | 43800/4.72 | Extracellular | Cytoplasmic | Cytoplasm |
| HX7 | gi|146318198 | pyruvate kinase [*Streptococcus suis* 05ZYH33] | 54596/5.12 | 55000/5.28 | Extracellular | Cytoplasmic | Cytoplasm |
| HX8 | gi|146318198 | pyruvate kinase [*Streptococcus suis* 05ZYH33] | 54596/5.12 | 55000/5.32 | Extracellular | Cytoplasmic | Cytoplasm |
| HX9 | gi|146319275 | S-adenosylmethionine synthetase [*Streptococcus suis* 05ZYH33] | 43062/5.01 | 53000/5.16 | Cytoplasmic | Cytoplasmic | Cytoplasm |
| HX10 | gi|146318011 | aspartyl/glutamyl-tRNA amidotransferase subunit B [*Streptococcus suis* 05ZYH33] | 53151/5.06 | 50000/5.22 | Cytoplasmic | Cytoplasmic | Cytoplasm |
| HX11 | gi|146318667 | ADP-glucose pyrophosphorylase [*Streptococcus suis* 05ZYH33] | 41382/5.01 | 46000/5.2 | Cytoplasmic | Cytoplasmic | Cytoplasm |
| HX12 | gi|146319824 | tRNA-specific 2-thiouridylase MnmA [*Streptococcus suis* 05ZYH33] | 42105/4.97 | 44000/5.1 | Cytoplasmic | Cytoplasmic | Cytoplasm |
| HX13 | gi|146320177 | fructose-bisphosphate aldolase [*Streptococcus suis* 98HAH33] | 31136/4.90 | 31000/5.13 | Cytoplasmic | Cytoplasmic | Cytoplasm |
| HX14 | gi|11612452 | elongation factor Tu [Streptococcus suis] | 29603/4.54 | 15000/5.25 | Cytoplasmic | Cytoplasmic | Cytoplasm |
| HX15 | gi|146318058 | hypothetical protein SSU05_0403 [*Streptococcus suis* 05ZYH33] | 31597/5.49 | 30000/5.78 | Unknown | Cytoplasmic | Cell membrane |
| HX16 | gi|253752618 | adenylosuccinate synthetase [*Streptococcus suis* SC84] | 47478/5.65 | 47000/5.94 | Cytoplasmic | Cytoplasmic | Cytoplasm |
| HX17 | gi|146319837 | Inosine 5'-monophosphate dehydrogenase [Streptococcus suis 05ZYH33] | 52743/5.61 | 55000/5.92 | Cytoplasmic | Cytoplasmic | Cytoplasm |
