# Supplementary material for: Pre-Absorbed Immunoproteomics: A Novel Method for the Detection of Streptococcus suis Surface Proteins
Source: PLoS One. 2011 Jun 21;6(6):e21234. doi: 10.1371/journal.pone.0021234 (PMC3119691; doi:10.1371/journal.pone.0021234)
Supplement: Table S2 — Distributions of the HX2 gene in Streptococcus sp. Strains. aThe serotypes of SS strains were confirmed by the agglutination test. N, nontypeable. (DOC) [file pone.0021234.s002.doc]

**Supporting Information**

**Table S2. Distributions of the *HX2* gene in *Streptococcus sp.* strains**

| **Strains** | **Serotype^a^** | **Origin** | **Presence of HX2 gene** |
| --- | --- | --- | --- |
| SH28 | 1 | Canada | ＋ |
| 26S1 | 1/2 | Canada | ＋ |
| HA9801 | 2 | China | ＋ |
| 700794 | 2 | ATCC | ＋ |
| T15 | 2 | Holland | ＋ |
| ZY050719 | 2 | China | ＋ |
| SS2-H | 2 | China | ＋ |
| SS2-Y | 2 | China | ＋ |
| SS2-YY060816 | 2 | China | ＋ |
| SS2-XT | 2 | China | ＋ |
| SS2-1 | 2 | China | ＋ |
| SS2-HA | 2 | China | ＋ |
| 19-2 | 2 | China | ＋ |
| JDE050802 | 2 | China | ＋ |
| HA050729-1 | 2 | China | ＋ |
| BB070118 | 2 | China | ＋ |
| SH040815 | 2 | China | ＋ |
| SS2-xy0811 | 2 | China | ＋ |
| 07-SS-rugao-01 | 2 | China | ＋ |
| 07-SS-rugao-02 | 2 | China | ＋ |
| 07-SS-nanjing-01 | 2 | China | ＋ |
| Hb1002 | 2 | China | ＋ |
| Hb1004 | 2 | China | ＋ |
| Hb1005 | 2 | China | ＋ |
| Hb1006 | 2 | China | ＋ |
| Hb1007 | 2 | China | ＋ |
| Hb1008 | 2 | China | ＋ |
| Hb1009 | 2 | China | ＋ |
| Hb1010 | 2 | China | ＋ |
| Hb1011 | 2 | China | ＋ |
| Hb1012 | 2 | China | ＋ |
| Hb1013 | 2 | China | ＋ |
| NH4 | 2 | China | ＋ |
| JA07109 | 2 | China | ＋ |
| LS091105 | 2 | China | ＋ |
| Hb1001 | 3 | China | ＋ |
| 090318 | 5 | China | ＋ |
| 090320 | 5 | China | ＋ |
| SH59 | 7 | China | ＋ |
| 0911-037m-x | 7 | China | ＋ |
| 0911-065m-1 | 7 | China | ＋ |
| 0911-065m-2 | 7 | China | ＋ |
| 090843 | 8 | China | ＋ |
| SH26 | 9 | China | ＋ |
| SH06 | 9 | China | ＋ |
| SH040917 | 9 | China | － |
| L89 | 9 | China | － |
| 55 | 9 | China | － |
| JX041226 | 9 | China | － |
| 2083 | 9 | China | ＋ |
| NJ-1 | 9 | China | ＋ |
| NJ-2 | 9 | China | ＋ |
| NJ-3 | 9 | China | ＋ |
| NJ-4 | 9 | China | － |
| NJ-5 | 9 | China | ＋ |
| NJ-6 | 9 | China | ＋ |
| SH13 | 10 | China | － |
| DL15 | 10 | China | － |
| Hb1016 | 12 | China | － |
| 8830 | 12 | Denmark | ＋ |
| NP4 | 12 | China | － |
| 091120 | 14 | China | ＋ |
| 0902-005m-x | 19 | China | － |
| 090202 | 20 | China | ＋ |
| 090306 | 25 | China | ＋ |
| Bs6 | 26 | China | － |
| HT68 | 26 | China | － |
| 090416 | 29 | China | ＋ |
| XZ202 | 31 | China | ＋ |
| 090339 | N | China | ＋ |
| 0911-057m-x | N | China | ＋ |
| Bs53 | N | China | － |
| Hb1015 | N | China | － |
| Hb1017 | N | China | ＋ |
| Hb1019 | N | China | ＋ |
| Streptococcus strains | | | |
| *S.equi subsp.zooepidemicus* | | | |
| 35246 | | ATCC | － |
| CY | | China | － |
| CC | | China | － |
| 555 | | China | － |
| 552 | | China | － |
| SH171 | | China | － |
| 1892 | | China | － |
| HN001 | | China | － |
| S.*iniae* | | | |
| 29177 | | ATCC | － |
| *S.agalactiae* | | | |
| HN-2 | | China | － |
| HN-6 | | China | － |
| HN-7 | | China | － |
| HN-9 | | China | － |
| CVCC 1886 | | China | － |

^a^ The serotypes of SS strains were confirmed by the agglutination test.

N, non-typeable.
